# An ancient FMRFamide-related peptide–receptor pair induces defence behaviour in a brachiopod larva

**DOI:** 10.1098/rsob.170136

**Published:** 2017-08-23

**Authors:** Daniel Thiel, Philipp Bauknecht, Gáspár Jékely, Andreas Hejnol

**Affiliations:** 1 Sars International Centre for Marine Molecular Biology, University of Bergen, Thormøhlensgate 55, 5006 Bergen, Norway; 2 Max Planck Institute for Developmental Biology, Spemannstraße 35, 72076 Tübingen, Germany

**Keywords:** Trochozoa, FMRFamide, neuropeptide receptor, defence behaviour, planktonic larva, brachiopod

## Abstract

Animal behaviour often comprises spatially separated sub-reactions and even ciliated larvae are able to coordinate sub-reactions of complex behaviours (metamorphosis, feeding). How these sub-reactions are coordinated is currently not well understood. Neuropeptides are potential candidates for triggering larval behaviour. However, although their immunoreactivity has been widely analysed, their function in trochozoan larvae has only been studied for a few cases. Here, we investigate the role of neuropeptides in the defence behaviour of brachiopod larvae. When mechanically disturbed, the planktonic larvae of *Terebratalia transversa* protrude their stiff chaetae and sink down slowly. We identified endogenous FLRFamide-type neuropeptides (AFLRFamide and DFLRFamide) in *T. transversa* larvae and show that the protrusion of the chaetae as well as the sinking reaction can both be induced by each of these peptides. This also correlates with the presence of FLRFamidergic neurons in the apical lobe and adjacent to the trunk musculature. We deorphanized the AFLRFamide/DFLRFamide receptor and detected its expression in the same tissues. Furthermore, the ability of native and modified FLRFamide-type peptides to activate this receptor was found to correspond with their ability to trigger behavioural responses. Our results show how FLRFamide-type neuropeptides can induce two coherent sub-reactions in a larva with a simple nervous system.

## 1. Background

Planktonic organisms have evolved different strategies to defend themselves from predation [1–4]. Morphological characters such as shells, spines or chaetae [5–7] and behaviours such as vertical migration, contraction, active fleeing or passive sinking [8–11] can help to cope with certain predators. This is especially true for ciliated larvae that do not possess an elaborated nervous system and face the challenge of remaining in the water column for dispersal while avoiding predation. The startle behaviour of several planktonic annelid and brachiopod larvae has often been described as a defence strategy, where they stop swimming and protrude long and pointed chaetae [12–16]. The co-occurring sub-reactions of spreading the chaetae and stopping swimming take place in spatially separated tissues: the internal trunk musculature and the ciliated apical edge, respectively. Both sub-reactions have to be coordinated within the framework of a larval nervous system. One mechanism to achieve coordination of different reactions could be the use of neuropeptides as signalling molecules. Neuropeptides are known to influence many behaviours and can be crucial in the regulation and coordination of spatially or temporally separated coherent sub-reactions. During insect ecdysis, for example, the eclosion hormone and ecdysis-triggering hormone both act as a form of master-regulator on different peripheral as well as central targets, and each coordinates several sub-reactions [17–20]. Another example is neuropeptide Y, which stimulates appetitive as well as consummatory ingestive behaviour in the Siberian hamster [21].

The influence of neuropeptides on the behaviour of trochozoan larvae has only been demonstrated in a few studies, which show that neuropeptides can trigger settlement and influence their ciliary-based locomotion [22–25]. One of the neuropeptides that has been shown to influence ciliary beating of different trochozoan larvae is FMRFamide [22,23,25]. FMRFamide-immunoreactivity is widely used as a marker for neural substructures in morphological studies [26,27]. Furthermore, while FMRFamide-related peptides (FaRPs) have been identified in many metazoans, their phylogenetic relationships are difficult to infer [28–30]. For the comparison of larval nervous systems, it is therefore of crucial interest to understand the functional role of a neuropeptide and its versatility to trigger larval behaviours.

While experimental studies in trochozoan larvae are limited, the physiological effect of FMRFamide-like peptides has been intensively investigated in adult trochozoans, where it has been found to have various effects on muscular activity [31–37]. Depending on the species, it can increase or decrease the heartbeat [31], cause contractions or relaxation of somatic muscles [32–34] or modulate the effects of classical neurotransmitters on somatic muscles [36,37]. Many immunohistochemical analyses on trochozoan larvae of different clades show FMRFamide-like immunoreactivity associated with muscles or ciliary bands [38–46], but experimental data are most often missing and functional studies are restricted to mollusc and annelid larvae [22,23,25]. Despite the recurring association of FMRFamide-like peptides with musculature in trochozoans, only one study has shown an effect of FMRFamide on the musculature of a trochozoan larva, which describes the induction of frequent contractions of the ciliated velum of *Tritia obsoleta* veliger larvae [22].

Since neuropeptides can act over longer distances [47], the localization of the neuropeptide receptor provides more information about the tissues actually affected than the peptide secreting cells that are labelled with the peptide antibodies. The majority of neuropeptide receptors are G-protein-coupled receptors (GPCRs), with a few exceptions like insulin receptors or peptide-gated ion channels [29,48,49]. Three different receptors for FMRFamide have been deorphanized in invertebrates so far. One is an FMRFamide-gated amiloride-sensitive Na^+^ channel (FaNaCh) that has been identified in molluscs [50–52]. The two other receptors belong to two different groups of neuropeptide GPCRs. One of these FMRFamide-GPCRs was identified in the fruit fly *Drosophila melanogaster* [53,54] and the other one in the annelid *Platynereis dumerilii* [49]. This stands in contrast to many cases in which homologous ligands also activate homologous receptors [49,55,56].

To expand the taxon sampling of functional neuropeptide studies in lophotrochozoans and to better understand the role of the widely used neuropeptide marker FMRFamide, we investigated the effects of an FaRP on the behaviour of a brachiopod larva. Recent research on brachiopods has revealed important insights in evolutionary developmental biology [57–59] and descriptions of their nervous system include the use of FMRFamide antibodies [60,61] and classical neuronal markers [62,63], as well as other molecular techniques [64]. Here, we show that the endogenous FLRFamide-like peptides induce the characteristic defence behaviour in the larvae of the brachiopod *Terebratalia transversa*, which consists of a downward sinking and the protrusion of their chaetae. Behavioural experiments and receptor deorphanization, in combination with immunohistochemistry, and *in situ* hybridization show that both sub-reactions can be specifically triggered by a single peptide acting via an ancient FaRP receptor. Together our results show how a single neuropeptide can trigger two coherent reactions and integrate evolutionary novelties such as trochozoan chaetae [57] into the *T. transversa* larval defence behaviour.

## 2. Material and methods

### 2.1. Collection and rearing of *Terebratalia transversa* larvae

Adult *T. transversa* (Sowerby, 1846) were collected in January 2015 and 2016 by dredging at approximately 50–100 m depth close to the University of Washington's Friday Harbor Laboratories, San Juan Islands, WA, USA. Larvae were obtained according to Stricker & Reed [65] by artificial fertilization and kept at 8–10°C. Two different types of larvae were used for the experiments: early larvae (2 days post-fertilization) before chaetal formation and late larvae (4–5 days post-fertilization) with clearly developed mantle lobes and long chaetae. For immunohistochemistry and *in situ* hybridization, larvae were relaxed in 7.8% MgCl_2_-6H_2_O in distilled water for 10–15 min, fixed in 4% methanol-free formaldehyde in seawater for 1 h, subsequently washed in PBS + 0.1% Tween and transferred into 100% methanol for storage at −20°C.

### 2.2. Bioinformatics

The previously published transcriptome of *T. transversa* (SRX1307070) was searched for peptide precursor and receptor candidates using BLAST. Publicly available FMRFamide-like precursor sequences from NCBI were used as reference sequences, and the resulting candidates were checked for signal peptides, cleavage sites and amidation sites. Neuropeptide precursor genes were also searched in the transcriptome of *Novocrania anomala* (SRX1343816). As reference sequences for the peptide receptor, previously published datasets [29,49] were used as well as transcriptomes of *Xenoturbella bocki* (SRX1343818), *Nemertoderma westbladi* (SRX1343819), *Meara stichopi* (SRX1343814) and *Halicryptus spinulosus* (SRX1343820) to obtain additional sequences. The candidates were compared using the software CLANS [66], with a *p*-value cutoff of 1 × 10^−70^. Sequences that were strongly connected in the cluster map were aligned with Clustal X v. 2.1 [67], non-conserved stretches were deleted manually, and the best fitting amino acid substitution matrix was determined with Modelgenerator v. 0.85 [68]. The final phylogenetic analysis was calculated with PhyML v. 3.0 [69] with 500 bootstrap replicates and visualized with Figtree v. 1.4.3 (http://tree.bio.ed.ac.uk/software/figtree).

### 2.3. Behavioural assays

**Figure 1.**
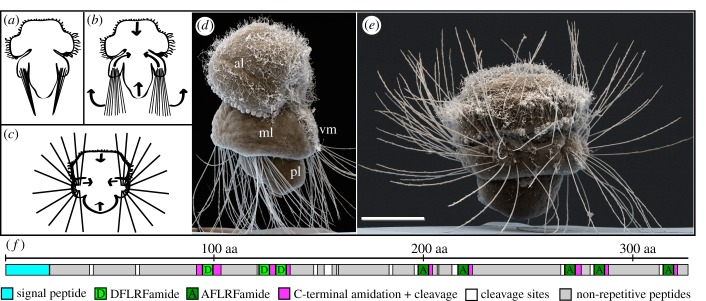
Defence reaction and FLRFamide prepropeptide of the *T. transversa* larvae. (*a*–*c*) sketch, (*d*,*e*) SEM photographs, anterior up, (*f*) sketch. (*a*) Larva in relaxed stance during normal swimming; (*b*) non-contracted larva that begins to spread its chaetae; (*c*) larva in defence stance with outspread chaetae; (*d*) non-contracted competent larva; (*e*) contracted competent larva with outspread chaetae; (*f*) schematic of *T. transversa* FLRFamide prepropeptide. al, anterior lobe; ml, mantle lobe; pl, pedicle lobe; vm, ciliated ventral midline. Scale bar, 50 µm.

The reaction of larvae to synthetic peptides (GenScript) that were predicted from the prepropeptide sequence were tested and compared with the reactions to peptides with non-native modifications. Freely swimming larvae were exposed to different concentrations of peptides in 4-well and 6-well plates (1 ml and 5 ml total volume per well, respectively), and their reactions were observed under a stereomicroscope. To determine the efficiency of the native and modified peptides, the larvae were tested for the necessary minimum concentration at which they fully contracted and spread their chaetae, using 30–100 larvae in each test. To get an estimation of the peptide concentration that was necessary to induce a complete contraction, the larvae were initially exposed to 50 nmol l^−1^ of the respective peptide and the concentration was then increased stepwise until the larvae fully contracted or a concentration of 50 µmol l^−1^ was reached as an upper cutoff. Larvae were considered fully contracted when their chaetae were spread in all directions (figure 1*c*) and when they did not further increase in contraction after an increase in peptide concentration, or after the use of the most sensitive peptide. To test the influence of the peptides on the vertical distribution of larvae in a water column, freely swimming larvae (50–100 per treatment) were exposed to peptides in transparent 4.5 ml cuvettes. About 60 s after addition of the peptides, the larvae were recorded with a digital camera (DMK 31AU03 camera, The Imaging Source) in a darkened box with artificial top-illumination. All experiments were repeated at least once with a different batch of larvae from another fertilization and the outcome was averaged. Control animals were exposed to DMSO only.

### 2.4. Receptor deorphanization

For the receptor deorphanization, the procedure was used as described by Bauknecht & Jékely [49]: full-length open reading frames of the receptor candidate sequences were amplified by PCR from cDNA of mixed larval stages. The forward primers included a 5′ attachment consisting of a spacer, an EcoR1 or BamH1 restriction site and a Kozak sequence and the reverse primers included a 5′ attachment consisting of a spacer and a Not1 restriction site (see the electronic supplementary material for primer sequences). The amplified products were cut with the corresponding restriction enzymes, cloned into pcDNA3.1(+) mammalian expression vector (Sigma-Aldrich), sequenced from both ends with a T7 forward and a bGH reverse primer, and transfected into CHO-K1 cells together with a calcium-sensitive luminescent apoaequorin-GFP fusion protein encoding plasmid (G5A) and a promiscuous Gα-16 protein encoding plasmid. After 2 days, Coelenterazine h (Promega, Madison, WI, USA) was added and incubated with the cells for 2 h. The luminescence response of the transfected cells was measured in a plate reader (BioTek Synergy Mx or Synergy H4, BioTek, Winooski, USA) over 45 s after addition of the neuropeptides. The response of the cells to 1 mM histamine was used as a general control in each plate. All measurements for the dose–response curves were made twice with different cell passages. Dose–response curves were calculated using Prism 6 (GraphPad, La Jolla, USA) and normalized against the upper plateau values (100% activation).

### 2.5. *In situ* hybridization

FLRFamide precursor and receptor sequences were amplified by PCR and cloned into pGEM T-easy vector (Promega) for *in vitro* transcription of DIG-UTP or DNP-UTP labelled RNA probes. For tropomyosin, a previously published clone [70] was used. The *in situ* hybridization protocol with an alternative hybridization buffer is published elsewhere [71]. The protocol from Hejnol [72] was adjusted with a proteinase K treatment (10 µg ml^−1^) of 8 min and with a postfixation in 3.7% formaldehyde + 0.2% glutaraldehyde in PBS + 0.1% Tween 20. The hybridization buffer contained 4 mol l^−1^ urea, 5× SSC, 1% dextran, 1% SDS, 50 µg ml^−1^ heparin, 50 µg ml^−1^ single-stranded DNA (no formamide). The signal was developed with the TSA Plus Cy3 or Cy5 kit (Perkin Elmer) or NBT/BCIP as a substrate and detected via fluorescence or NBT/BCIP reflection [73] with a Leica SP5 confocal laser-scanning microscope.

### 2.6. Immunohistochemistry

Customized polyclonal antibodies were raised in rabbits against CFLRFamide, coupled via a disulfide bridge to Keyhole limpet hemocyanin (GenScript^®^). Co-staining was either done with mouse anti-acetylated α-tubulin (Sigma, T6793) or mouse anti-actin (Seven Hills Bioreagents, LMAB-C4) antibodies. For the staining procedure, the protocol of Conzelmann & Jékely [74] was used with the following adjustments: proteinase K treatment (10 µg ml^−1^) was done for 3–5 min, and after the proteinase inactivation step with glycine (2 mg ml^−1^) the samples were incubated for 2–4 h in PBS + 0.5% TritonX. Primary antibodies were incubated over three nights at 4°C and washed for 4–6 h with at least 10 changes of washing medium. Secondary antibodies (Alexa 555 goat anti-rabbit and Alexa 647 goat anti-mouse) were incubated overnight and an additional secondary antibody (Alexa 488 goat anti-rat) without corresponding primary antibody was included to test and subtract unspecific staining. After washing the secondary antibodies for 4–6 h with at least 10 changes of buffer, specimens were transferred into methanol and mounted in Murray's clear (2 : 1 parts benzyl benzoate : benzyl alcohol).

## 3. Results

### 3.1. The endogenous neuropeptides DFLRFamide and AFLRFamide trigger the defence behaviour of *Terebratalia transversa* larvae

During normal swimming, the chaetae of competent *T. transversa* larvae rest against their pedicle lobe with their tips forming a bundle (figure 1*a*). When the larvae get disturbed (e.g. mechanical irritation with a pipette tip), they stop swimming, sink down slowly and exhibit a defensive stance by lengthwise contraction of their body to spread the four bundles of chaetae outwards (figure 1*b,c*,*e*; electronic supplementary material, Video S1). At maximal contraction, the larvae spread their chaetae in all directions to surround their soft body (figure 1*c*,*e*).

We identified an FLRFamide prepropeptide sequence in the transcriptome of *T. transversa*. The FLRFamide precursor contains a signal peptide, three copies of DFLRFamide and five copies of AFLRFamide, partially separated by intermediate sequences (figure 1*f*; see also the electronic supplementary material for colour-coded amino acid sequence). When we exposed larvae to synthetic FLRFamide, they contracted lengthwise, spread out their chaetae and sank down slowly. Both predicted peptides, DFLRFamide and AFLRFamide, caused the same behaviour. When exposed to 50–100 nmol l^−1^ DFLRFamide, all larvae showed initial signs of contraction, indicated by their chaetae bundles being slightly fanned out while still pointing in a posterior direction (figure 1*b*,*d*). About half of the larvae continued swimming while the other half started to sink slowly towards the bottom. An increase in peptide concentrations led to an overall increase in contraction, resulting in a stronger spreading of the chaetae and more larvae sinking. A maximum contraction of all larvae, with their body being completely surrounded by chaetae, was observed at concentrations of 500–750 nmol l^−1^ DFLRFamide. When we removed the peptides by exchanging the medium with fresh seawater, all larvae returned to normal swimming behaviour. Continuous exposure for about 2 h led to a desensitization and the larvae resumed normal swimming without removing the peptides. When the larvae were desensitized by continuous exposure to DFLRFamide, they also became insensitive to AFLRFamide and vice versa. Desensitized larvae still showed an initial contraction when they were disturbed, but it seemed like they had to be disturbed more harshly than non-desensitized larvae. Larvae that were exposed to DFLRFamide concentrations below the threshold that is able to induce a defensive behaviour (50 nmol l^−1^) seemed to be more sensitive than untreated ones and already contracted when slightly disturbed. However, we were not able to quantify the necessary strengths of disturbance. We also tested orthologues of AFLRFamide/DFLRFamide on late chaetous larvae of *P. dumerilii* and *Novocrania anomala* (FMRFamide and YMRFamide, respectively; see the electronic supplementary material for *N. anomala* precursor sequence), but even concentrations above 50 µmol l^−1^ did not induce any defence reaction.

Taken together, we found that *T. transversa* larvae show a sustained behaviour which is similar to their startle response when we exposed them to one of the neuropeptides encoded on the endogenously expressed FLRFamide precursor.

### 3.2. FLRFamide causes sinking of larvae independent from the protrusion of their chaetae

**Figure 2.**
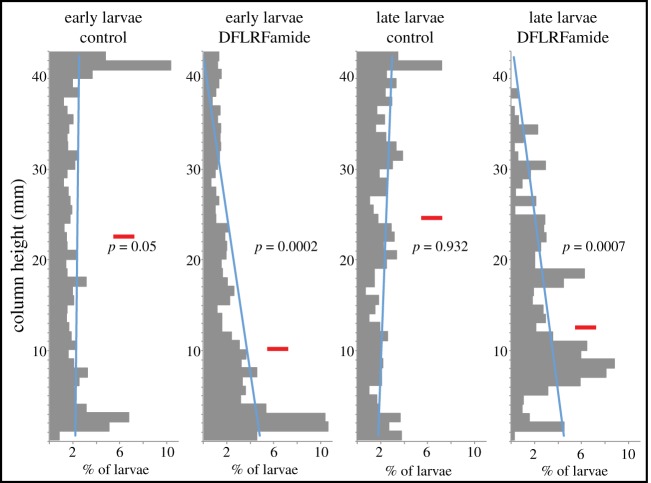
Influence of FLRFamide on the vertical distribution of early and late larvae in a water column. Horizontal red bar shows average level of swimming height, *p*-values are calculated for difference in distribution of larvae in upper versus lower half of the column (two-tailed, unpaired *t*-test), blue line is the estimated trend line (not statistically supported). Distribution was measured over a period of 5 s, about 1 min after exposure to 5 µmol l^−1^ DFLRFamide.

One part of the defence behaviour of *T. transversa* larvae is a slow downward sinking. A similar reaction can already be observed in early larvae before they develop long chaetae. Owing to the shape of the larvae and the lack of a clear restriction of the prototroch, it was not possible to directly record the ciliary beating. However, because *T. transversa* larval locomotion is purely driven by ciliary beating, we hypothesize that FLRFamide influences the ciliary movement. To measure the swimming behaviour in an unbiased manner, we recorded the position of freely swimming larvae in vertical columns and compared it to the position of larvae after exposure to DFLRFamide. To test the possibility that the sinking is caused by an increase in the water drag due to the protruded chaetae, we also recorded early larvae that already express FLRFamide in the apical lobe (electronic supplementary material, figure S1*d*) but do not possess long chaetae yet (electronic supplementary material, figure S1*e*). Both stages showed a sinking behaviour that shifted the distribution of the larvae in the water column downwards, compared to the controls (figure 2). Early larvae kept swimming more freely close to the bottom, whereas late larvae usually stayed at the bottom and moved only very slowly forward.

### 3.3. Modified peptides trigger the defence behaviour at different concentration thresholds

We tested at which concentration modified peptides induce the contraction that leads to the erection of the chaetae, with 50 µmol l^−1^ as a cutoff for the maximum concentration (table 1; electronic supplementary material, table S1). The larvae were most sensitive to DFLRFamide and showed full contraction (figure 1*c*,*e*), sinking and very slow movements on the bottom of the dish at concentrations between 500 and 750 nmol l^−1^ (batch dependent). Further increasing of the concentration did not lead to an obvious increase in the reaction. AFLRFamide was slightly less effective by triggering full contraction of all larvae between 1 and 1.5 µmol l^−1^. The reduced peptide sequence FLRFamide was effective at 3 µmol l^−1^. Changing the amidated C-terminal phenylalanine to an amidated tryptophan reduced the effectiveness by about 10-fold, with a minimum necessary concentration of 7.5 µmol l^−1^ for DFLRWamide or 20 µmol l^−1^ for AFLRWamide. Changing the C-terminal phenylalanine to the non-aromatic leucine only led to a very weak contraction (similar to figure 1*b*) in some of the larvae at 50 µmol l^−1^ DFLRLamide, whereas 50 µmol l^−1^ AFLRLamide gave no reaction at all. Reducing the sequence to the three C-terminal amino acids, LRFamide, also did not lead to any contraction, and nor did any of the other endogenous peptides that we tested. (A list of tested peptides is given in the electronic supplementary material, table S1.) The overall most effective versions were the ones that are encoded on the pro-peptide sequence, DFLRFamide and AFLRFamide. The reduced peptide FLRFamide was slightly less effective and a modification of the amidated C-terminus reduced the effectiveness even more.

**Table 1.** Necessary peptide concentrations to evoke larval defence stance compared to EC_50_ values of receptor activation.

peptide	necessary concentration to induce full contraction (µmol l^−1^)	EC_50_ receptor assay
DFLRFamide	0.625	26.5 nmol l^−1^
AFLRFamide	1.5	12.4 nmol l^−1^
FLRFamide	3	33.2 nmol l^−1^
DFLRWamide	8.75	0.9 µmol l^−1^

### 3.4. Identification of the *Terebratalia transversa* FaRP receptor

**Figure 3.**
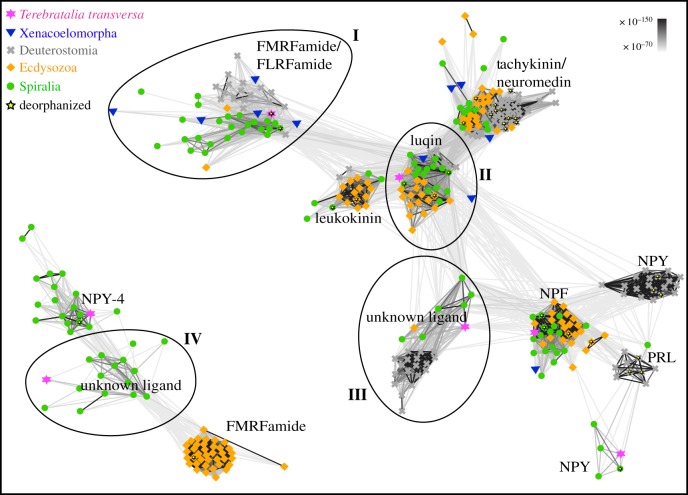
Clustermap of metazoan neuropeptide GPCRs related to FMRFamide receptors. Connections correspond to blastp connection with *p*-values of less than 1 × 10^−70^. Groups that include the receptors I–IV that were tested for activation by FLRFamide are encircled. NPY, neuropeptide Y; NPF, neuropeptide F; PRL, prolactin releasing peptide.

Based on BLAST e-value similarities and cluster analyses, we tested four receptor candidates for their activation by FLRFamide (figure 3). One candidate belongs to a cluster of receptors that includes the deorphanized *P. dumerilii* FMRFamide receptor [49] with related sequences in all major bilaterian groups including Xenacoelomorpha (figure 3 ‘I’; electronic supplementary material, figure S2). The second candidate belongs to the luqin receptors (figure 3 ‘II’; electronic supplementary material, figure S2). The third candidate belongs to a group of related receptors with unknown ligand (figure 3 ‘III’; electronic supplementary material, figure S2), and the fourth one belongs to a group that shows similarities with the *Drosophila melanogaster* FMRFamide and the *P. dumerilii* NPY-4 receptors and is not related to the other three receptors (figure 3 ‘IV’). Transcriptome searches for an FMRFamide-gated ion channel (FaNaCh), which has been identified in molluscs, did not reveal any orthologues in *T. transversa*, even when using FaNaCh orthologues that were identified in the brachiopods *Lingula anatina* and *Novocrania anomala*.

**Figure 4.**
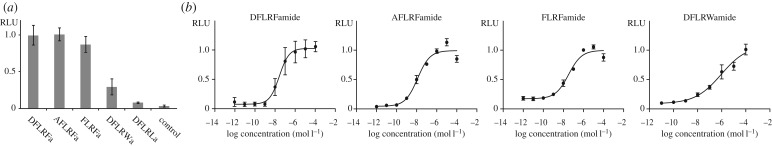
Luminescence response of *T. transversa* FLRFamide receptor expressing CHO-K1 cells to different peptides. (*a*) Relative luminescence of *T. transversa* FLRFamide receptor expressing cells after exposure to different peptides with a fixed concentration of 1 µmol l^−1^. (*b*) Dose–response curves of *T. transversa* FLRFamide receptor expressing cells to different concentrations of DFLRFamide, AFLRFamide, FLRFamide and DFLRWamide. Luminescence values are given relative to maximum luminescence (max = 1). RLU relative luminescence.

To test whether FLRFamide is the ligand of one of these receptors, we tested their activation by DFLRFamide in transfected CHO-K1 cells. Only the candidate that is related to the *P. dumerilii* FMRFamide receptor was activated by 1 µmol l^−1^ DFLRFamide, but none of the other tested receptors. We therefore called this receptor the *T. transversa* FLRFamide receptor. As DFLRFamide triggered the defence stance at concentrations below 1 µmol l^−1^ in the behavioural assay, we did not test the negative GPCRs at higher peptide doses. We further compared the luminescence response of FLRFamide receptor expressing CHO-K1 cells to 1 µmol l^−1^ DFLRFamide, AFLRFamide, FLRFamide, DFLRWamide and DFLRLamide (figure 4*a*). The two native forms DFLRFamide and AFLRFamide led to the highest luminescence, followed by FLRFamide in a similar range. DFLRWamide gave a strongly decreased luminescence and the values of DFLRLamide were barely higher than the negative control. Dose–response curves were recorded for DFLRFamide, AFLRFamide, FLRFamide and DFLRWamide (figure 4*b*; electronic supplementary material, figure S3) and EC_50_ values (half maximal effective concentration) were determined for each peptide. AFLRFamide showed the lowest EC_50_ value (1.24 × 10^−8^ mol l^−1^), the EC_50_ for DFLRFamide was about two times as high (2.65 × 10^−8^ mol l^−1^), the one for FLRFamide was about three times higher (3.32 × 10^−8^ mol l^−1^) and the one for DFLRWamide was the highest of all tested peptides (9.06 × 10^−7^ mol l^−1^). The EC_50_ values are listed in table 1, together with the concentrations necessary to trigger the defence stance in the behavioural assay.

After we deorphanized the FLRFamide receptor, we tested its phylogenetic relationship to the receptors that showed connections in the cluster analysis (figure 3 ‘I–III’). We did not include the unrelated *T. transversa* orphan receptor that is related to the insect FMRFamide and *P. dumerilii* NPY-4 receptors (figure 3 ‘IV’). As seen in the cluster analysis, the *T. transversa* FLRFamide receptor is directly related to the *P. dumerilii* FMRFamide receptor and several orphan receptors of other trochozoans (electronic supplementary material, figure S2). Orthologues to these trochozoan FMRFamide/FLRFamide receptors were found in the insect *Nilaparvata lugens*, the hemichordate *Saccoglossus kowalevskii*, and the xenacoelomorph *Meara stichopi*. Further related GPCRs include orphan receptors from the cephalochordate *Branchiostoma floridae*, the ghost-shark *Callorhinchus milii*, and the xenacoelomorphs *M. stichopi* and *Nemertoderma westbladi*. All of these receptors form a fully supported group of neuropeptide GPCRs with homologues in all major bilaterian clades that are well separated from other neuropeptide GPCR groups (electronic supplementary material, figure S2).

In summary, we discovered that the *T. transversa* FLRFamide receptor belongs to an ancient neuropeptide receptor group and is efficiently activated by the two peptides AFLRFamide and DFLRFamide that are encoded on the *T. transversa* prepropeptide sequence*.*

### 3.5. *In situ* hybridization and immunohistochemistry show localization of peptide receptor in trunk musculature and apical prototroch region

**Figure 5.**
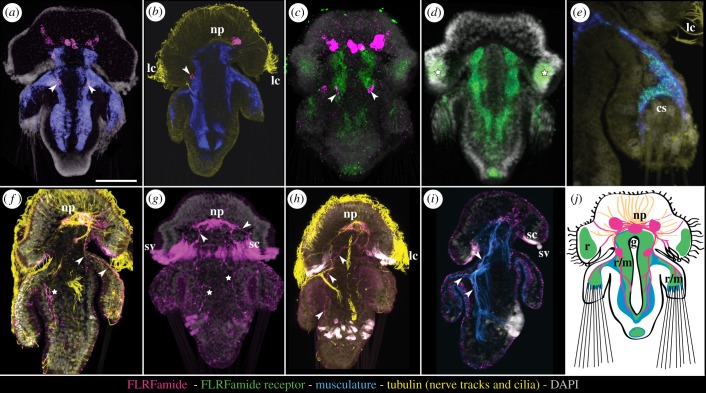
*In situ* hybridization and immunostaining of *Terebratalia* FLRFamide, *Terebratalia* FLRFamide receptor, musculature and tubulin. (*a–e*) *in situ* hybridization; (*f*–*i*) immunohistochemistry; (*a*,*c*,*d*,*e*,*g*,*j*) front view; (*b*,*f*,*h*,*i*) side view, ventral side left. (*a*,*b*) *FLRFa* and *tropomyosin* expression, arrows show *FLRFa* expression in mantle lobe. (*c*) *FLRFa* and *FLRFa receptor* expression, arrows show *FLRFa* expression in mantle lobe. (*d*) *FLRFa receptor* expression, stars show expression underneath prototroch. (*e*) *tropomyosin* and *FLRFa receptor* co-expression around chaetae sacs. (*f*) FLRFa and tubulin staining, star shows branching of FLRFa-positive nerves inside ventral trunk area, arrows show branching of dorsal FLRFa-positive trunk-nerve towards chaetae sacs. (*g*) FLRFa staining, stars show branching of FLRFa-positive nerves inside dorsal trunk area, arrows show projections into secretory cells underneath the prototroch. (*h*) FLRFa and tubulin staining, arrows show nerve projecting from neuropile into ventral part of the trunk. (*i*) FLRFa and actin staining, arrows show lining of the musculature by FLRFamidergic nerves, projecting into mantle and posterior part of the trunk. (*j*) Schematic drawing of FLRFamidergic cells and nerves, FLRFa receptor and musculature. cs, chaetae sacs; g, gut; lc, locomotory cilia of the prototroch; m, musculature; np, neuropile; r, receptor; sc, secretory cells; sv, secretory vesicles. Colour code is indicated at the bottom of the figure plate: magenta, FLRFamide ((*a*,*b*,*c*) precursor expression, (*f*–*i*) peptide antibody staining); green, FLRFamide receptor; blue, musculature ((*a*,*b*,*e*) tropomyosin expression, (*i*) actin antibody staining); yellow, nerve tracks and cilia; grey, DAPI. Scale bar, 50 µm.

The FLRFamide precursor has several expression domains within the apical lobe around the neuropile, and two domains on the ventral side at the anterior border of the mantle lobe (figure 5*a*–*c*; electronic supplementary material, figure S1*b*,*c*). The number of domains in the apical lobe varies between three and five (figure 5*a*,*c*) and each domain consists of approximately three to seven cells. The combined *in situ* hybridization with *tropomyosin* as a marker for the musculature shows that the FLRFamide precursor expression in the mantle lobe is adjacent to the ventral side of the trunk musculature (figure 5*b*). The FLRFamide receptor is expressed in a left and a right stripe in the trunk musculature (figure 5*c*,*d*; electronic supplementary material, figure S1*a*) as well as in the musculature that projects towards and surrounds the chaetae sacs (figure 5*e*; electronic supplementary material, figure S1*a*). Apart from the expression in the musculature, the receptor is also expressed in the apical lobe in a broad stripe underneath the ciliated prototroch (figure 5*c*,*d*; electronic supplementary material, figure S1*a*).

Since *in situ* hybridization only reveals where the peptide precursor is expressed, we used antibody stainings to visualize the nerves that secrete the active peptides. The customized FLRFamide antibody revealed immunoreactive longitudinal nerves that project from the apical neuropile (figure 5*f*,*g*,*h*,*i*) pairwise along the ventral (figure 5*h*) and dorsal (figure 5*f*) side into the trunk after branching off into the mantle towards the chaetae sacs at the border of the apical lobe and the mantle lobe (figure 5*f*,*h*,*i*). The nerves in the trunk are branching off strongly on the ventral side (figure 5*f*,*g*) and are, at least partially, directly adjacent to the musculature (figure 5*i*). The neuropile shows generally strong FLRFamide immunoreactivity with some nerves projecting towards the apical ciliary band (figure 5*f*) and into the secretory cells that continue into secretory vesicles outside the apical lobe underneath the prototrochal region (figure 5*g*,*i*). The secretory cells and vesicles themselves are prone to antibody trapping so no statement can be made as to whether they in fact contain FLRFamide (compare figure 5*g* without background subtraction and figure 5*f*,*h*,*i* with background subtraction in secretory cells and secretory vesicles).

## 4. Discussion

### 4.1. FLRFamide triggers two coherent reactions via an ancient FaRP receptor

The receptor deorphanization and phylogenetic analysis shows that the *Terebratalia* FLRFamide receptor belongs to the ancient FaRP-GPCR group with closely related trochozoan GPCRs that include the deorphanized *P. dumerilii* FMRFamide receptor [49] and related orphan GPCRs in all major bilaterian groups. The comparable sensitivity to different peptide modifications of the larvae in the behavioural assay and the EC_50_ values of the receptor cell assay suggests that the larval response is triggered via the FLRFamide receptor. The expression of the FLRFamide receptor in the longitudinal trunk musculature and the musculature adjacent to the chaetae sacs in the mantle supports a direct mode of signalling, whereby FLRFamide is able to trigger the protrusion of the chaetae by inducing a muscle contraction. The expression of the receptor in a broad stripe underneath the ciliary band and the sinking of early and late larvae, independent of the presence or the absence of chaetae, also support a direct effect of FLRFamide on the ciliated cells to induce the sinking behaviour. While a direct influence of FMRFamide on the ciliary movement of trochozoan larvae has already been suggested before [23,25], the combination of this reaction with the muscular contraction observed in the *T. transversa* larval startle response consists of two different behavioural actions. In the context of the natural *T. transversa* defence behaviour, the two FLRFamide-like peptides are probably not the main neurotransmitter of their peptidergic neurons, as larvae that are desensitized to AFLRFamide/DFLRFamide are still able to show a defensive stance, although it seems like the stress level has to be increased. A possible explanation for the role of FLRFamide might be that it acts as a co-transmitter, to modify or support the signals in the different tissues that are necessary for this defence behaviour.

### 4.2. The advantage of coherent sub-reactions during *Terebratalia transversa* defence behaviour and their control by a single peptide

Neuropeptides are considered to be ancient signalling molecules that are used in complex as well as simple nervous systems and are even present in *Trichoplax* which lacks neurons entirely [29,75]. There are a few examples of complex behaviours that involve coherent sub-reactions like insect ecdysis or feeding, which are known to deploy single neuropeptides to act on several targets as a form of master-regulator [17–21]. When a single neuropeptide is able to trigger or support the erection of chaetae and sinking, it might be involved in coordinating the startle reaction independently from a direct neuronal wiring between these two structures.

While many zoo-planktonic organisms escape potential predators by a sudden increase in velocity, some species have been observed to use passive sinking as an efficient escape strategy instead [3,76]. Passive sinking seems efficient for slow animals to escape quicker predators such as copepods that do not detect their prey by vision but by sensing water disturbance [3,11,77]. It has been described that other brachiopod and annelid larvae seem to have a similar startle behaviour as *T. transversa* [12–16]. Direct observations showed that small fish spit out *Sabellaria* larvae with their spines erected [15] and experimental data showed that *Sabellaria* larvae with long chaetae have a higher survival rate when exposed to different predators compared with younger larvae without chaetae [14]. The combination of a passive sinking behaviour while actively erecting chaetae might increase the chance to escape different predators when compared with either behaviour alone. The increased water drag due to the erected chaetae would probably also make an active fleeing inefficient.

Our results demonstrate a case in which a single receptor–ligand pair can trigger two coherent reactions that integrate evolutionary novelties such as trochozoan chaetae [57] and ancient traits such as ciliary-based locomotion [78] into the *T. transversa* larval startle behaviour.

### 4.3. The involvement of a specific neuropeptide in certain behavioural traits is not necessarily conserved during evolution

While the FaRP receptor–ligand pair in *P. dumerilii* and *T. transversa* is conserved, the involvement of FaRPs in trochozoan larval behaviour seems to vary. Several studies on trochozoan larvae have shown that FMRFamide-like immunoreactive nerves can be associated with different structures in a single animal and often include a combination of the apical organ, ciliary bands and the musculature [38–41,43,79]. In this context, it is also important to mention that the antibodies against FMRFamide that are commonly used in morphological studies can cross-react with other peptides ending in RFamide, even within the same species [80,81]. Inter-species comparisons and homologizations of such labelled neurons, especially across larger evolutionary distances, are therefore problematic. Only a few experimental studies exist on the effect of FMRFamide on trochozoan larvae and those focus on the regulation of the ciliary-based locomotion, which ultimately influences the vertical swimming direction [22,23,25]. These experiments on trochozoan larvae showed a taxon-specific up- or downregulation of the ciliary beating or the ciliary arrests. The ciliary beating alone, however, can be influenced by more than one peptide in a single species [23], as different neuronal circuits seem to trigger similar or opposing effects of the same effector organ and might thereby fine-tune the reaction, based on different neuronal inputs. Studies on adult trochozoans show diverse effects of FMRFamide on various muscles [32–35] and further taxon-specific functions such as osmoregulation [82], chromatophore expansion [83] or suppression of salivary gland activity [84]. Even experiments on different adult bivalve species showed species specific up- or downregulation of the heartbeat by FMRFamide [31]. The seemingly ubiquitous presence of FaRPs in trochozoan species with various taxon-specific effects and association with different tissues suggest that the FMRFamide-like peptides proved to be generally useful as a regulatory signalling system and were probably redeployed several times during trochozoan evolution, rather than having a strictly conserved role that is always associated with similar behavioural traits. The observation that the presumed orthologues in the annelid *P. dumerilii* and the brachiopod *N. novocrania* (FMRFamide and YMRFamide, respectively) do not trigger their respective defence behaviours supports the hypothesis that the involvement of a specific neuropeptide in similar behavioural traits is not necessarily conserved.

## Supplementary Material

Electronic Supplementary Information

## Data accessibility

Sequences of the here described genes and the accession numbers of publically available sequences that were used in this study are listed in the electronic supplementary material. Nucleotide sequences of the FLRFamide precursor and FLRFamide receptor are available on GenBank (MF543007, MF543008). Additional material with confocal stacks of stained larvae and videos of the influence of DFLRFamide on swimming larvae in the vertical assay is available at https://doi.org/10.6084/m9.figshare.c.3830605.v1. The original datasets generated and analysed during this study are available from the corresponding author on reasonable request.
