# Supplementary material for: An ancient FMRFamide-related peptide–receptor pair induces defence behaviour in a brachiopod larva
Source: Open Biol. 2017 Aug 23;7(8):170136. doi: 10.1098/rsob.170136 (PMC5577450; doi:10.1098/rsob.170136)
Supplement: Electronic Supplementary Information [file rsob170136supp1.pdf]

Electronic Supplementary Material

An ancient FMRFamide-related peptide-receptor pair induces defence behaviour in a brachiopod larva

Daniel Thiel, Philipp Bauknecht, Gáspár Jékely and Andreas Hejnol

Open Biology, 7: 170136

http://dx.doi.org/10.1098/rsob.170136

Table S1: Peptides that were tested in *Terebratalia transversa* larvae and the necessary concentrations to induce defense stance.

**peptide**	**batch 1**	**batch 2**
**DFLRFamide***	500 nM for full contraction	750 nM for full contraction
**AFLRFamide***	1 µM for full contraction	2 µM for full contraction
**FLRFamide****	3 µM for full contraction	3 µM for full contraction
**LRFamide****	no contraction at 50 µM	no contraction at 50 µM
**DFLRWamide**	7.5 µM for full contraction	10 µM for full contraction
**AFLRWamide**	20 µM for full contraction	20 µM for full contraction
**DFLRYamide**	50 µM for full contraction	50 µM for full contraction
**AFLRYamide**	weak contraction in about 50% of the larvae at 50 µM	no contraction at 50 µM
**DFLRLamide**	weak contraction in about 50% of the larvae at 50 µM	no contraction at 50 µM
**AFLRLamide**	no contraction at 50 µM	no contraction at 50 µM
**YMRFamide*****	10 µM for full contraction	15 µM for full contraction
**NSDGLamide***<br>**TDKCVPVYamide***<br>**AAKAPSSSamide***<br>**CYLYDCINamide***<br>**MDPSQFGYGIamide***<br>**YSLDGIGSGLIamide***<br>**LSDYYAWAAQTRLamide***<br>**GWamide***<br>**RGWamide(*)**<br>**LGWamide****<br>**FIamide****<br>**KPIIYEamide****<br>**WQGMKMWamide*****	no contraction at 50 µM	no contraction at 50 µM

* peptides predicted from *T. transversa* prepropeptide sequences
** shortened versions of peptides predicted from *T. transversa* prepropeptide sequences
*** peptides predicted from *Novocrania anomala* prepropeptide sequences

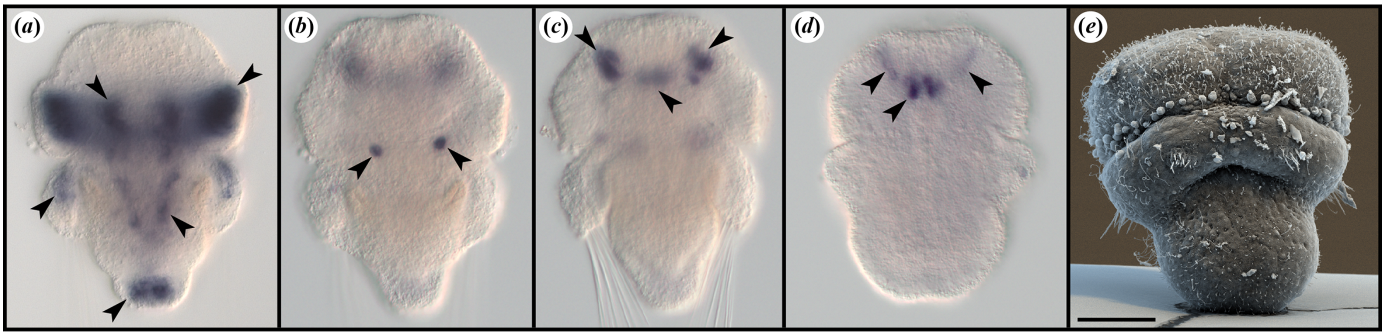

Figure S1: FLRFamide peptide and receptor expression in *T.transversa* larvae. **A-C** late larvae, *in situ* hybridization. Arrows indicate expression domains. **A** FLRFamide receptor expression. **B** ventral FLRFamide expression between apical and mantle lobe. (Same specimen as in C, with a focus on the ventral side.) **C** FLRFamide expression in apical lobe. **D** early larva with FLRFamide expression in apical lobe. **E** SEM picture of early larva. Scale bar = 30 µm.

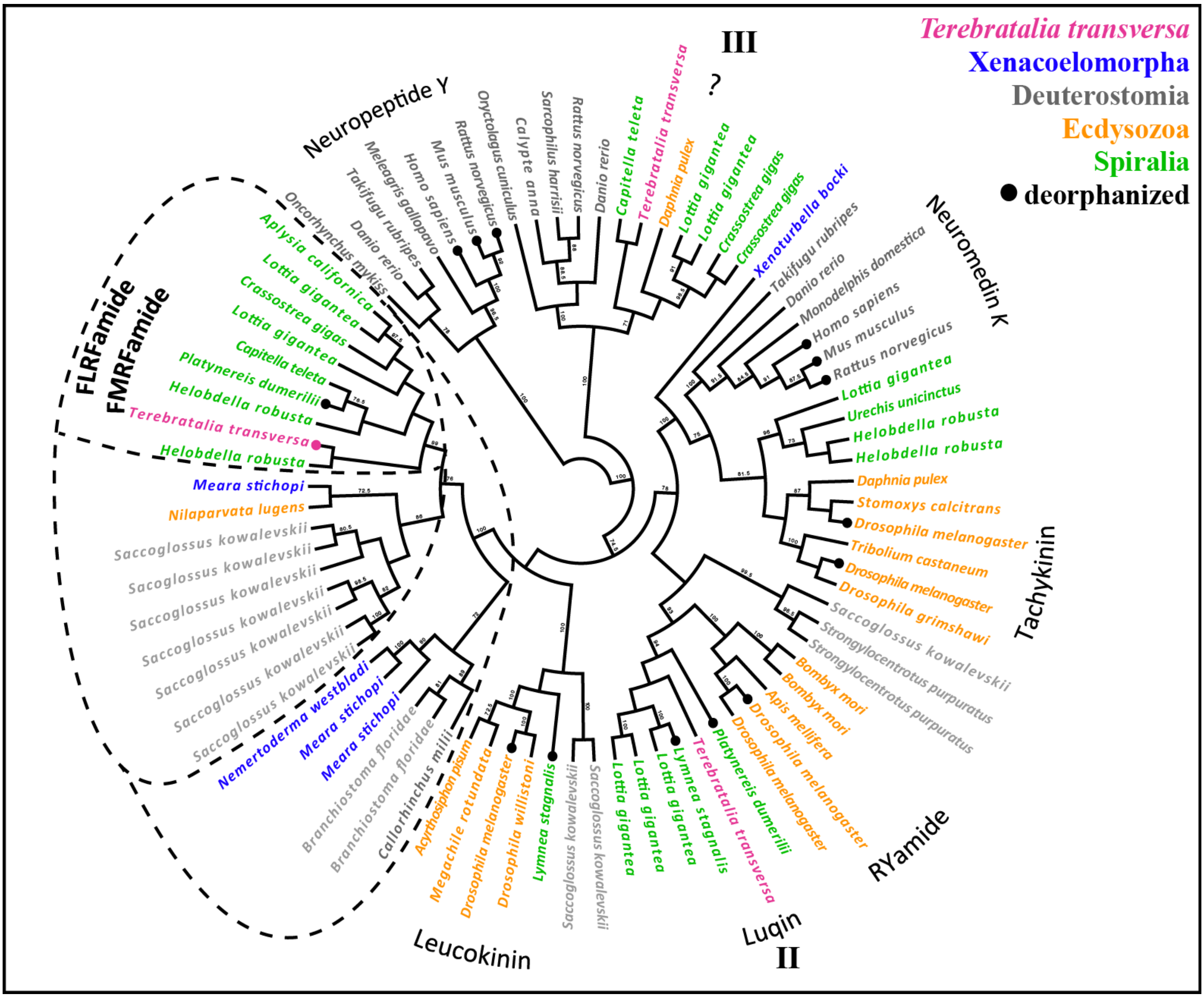

Figure S2: Phylogeny of the *T. transversa*-FLRFamide receptor. Cladogram of neuropeptide GPCRs that showed connections in the clustermap to the *T. transversa* FLRFamide receptor (Figure 3). The dashed lines indicate receptor groups related to the *Terebratalia* FLRFamide receptor. Branches with filled circle at the end indicate a receptor that was deorphanized in a previous study.

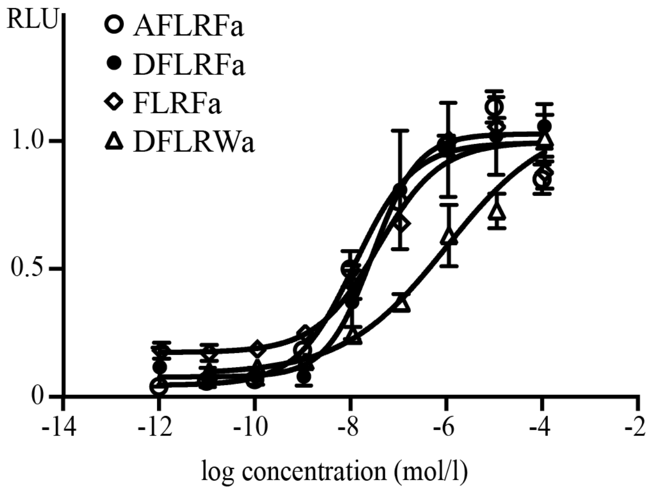

Figure S3: Combined dose-response curves of the *T. transversa* FLRFamide receptor to AFLRFamide, DFLRFamide, FLRFamide and DFLRWamide. RLU relative luminescence.

Terebratalia transversa FLRFamide prepropeptide sequence

MNRSVLLVAVVASFLLDHSVGIYTSRYINPCSPWLKSTHFRKLCDESFWPSYGSQEKDMIPYKRGFFGGGGEYGEPEG
LSSFVDEPYRGTIGKRDFLRFGKRRYDDNERSDQLEALNRVTRDFLRFGKRDFLRFGRSYNLRGYDSDNKRKHMRKRR
SIKNESDKPSSIESNDVYNKGALLRSDKKSMSAAKARDIKRAFLRFGRLQKEYQQQDRKRAFLRFGKNTGINLDNRPT
SDTKTETNSETDNGSAKTVTNDHVIDNNVDSKRAFLRFGKKCASCKRAFLRFGKSEKNLISSSNKSMKSSPLFGKEHT
KRAFLRFGKSVNRE*

Novocrania anomala YMRFamide prepropeptide sequence

MAKEAEMALLAAVLVIQLMVIPSGASQEKEAAKNMEFNQLRDDLSKLQQQLDDIKVLTAEVPEDKRFFDTPRYLTKRY
YLRFGRGIVDPYPYEHPSDAVDGGFFDKPHYGFNKRHYLRFGRGIIDSNPHGYPSNALEGAMADGFEKNKRYLRFGRS
VDRYVETPETHHLSKRSVKQKTPENGKTKEISPPKKETIKDKRYMRFGKKSIEDEKRYMRFGKKSDEDAQKRYMRFGK
KSDNNEEKRYMRFGKKSDDEKEKRYMRFGKKDESPDE

signal peptide - cleavage site - C-terminal amidation site – predicted FLRFamide like neuropeptides

Terebratalia GPCR sequences:

Terebratalia transversa FLRFamide receptor (deorphanized) (R1)

MAAAKEFPGIIRKRESYFKPVYIVNDKLLLNTTTTIRATTGLPLGHDTTMVTSMELNCTLPNCTNTTNDTTNGGSPIPLADSAQA
LIITFYTLAIILAAFGNILAIIIFSTGRRSRGDLRTYLLNLALADLAMSVFCIPFTFPTIIYHYWQFGSAMCTIVLFLQTATVVVS
VSTNMAIGIDRFLAVTFPLRSRASRKQKVVKRIVVVVIWCLSFLLASPNFVVAQTVDLGNGYYQCTEKWPGGKQPKLIFGIFILIF
TYIIPLVILLTTYGIIAMKLWRRQAPGEANRARDEAQLQSKRKVIKMLFTIVLLFGVCWLPLHTFINVLDFNPELAQNADQRILEI
IYICFHWLAMSNSFQNPIIYGFLNDNFRADFWDLIFVCLPCCSKAKYFKNHRRMSYTRSPANRWQQSSFSHLERRASARPLRNGST
SSSSDTDKHNKFNSFARSKTDTALLGVHEPSKGRTFIKRNGAKRSKQPMALVVPTVTYNSRDNDVTSREMEKLLEGIEPDTSEKTN
SIK*

Terebratalia transversa orphan luqin GPCR homolog (R2)

FISMFSAITVLSITGNVLVCFAVLRNQTMRSSSYFFILNLAVSDILMATMCIPFTFVANVLLDWWPFGHVMCPLVNFLQAMAVFLSAFTLIVISL
DRYVAIIFPLKARLTTKQIKVVIGIVWCCAVIPPIPIAVYGRVIRYVGRAYCQEIWTDNNNRFVYSLCILGLQFFVPLMVLVYTYIRIGIVIWGK
KFPGEAEYNRDQRMATSKRKMVKMMMMVVIMYIICWLPYHCITIHGSIDDTFYDEEHAPTLWVFAYWLAMSNSCCNPLIYFYMNSKFRRSFKKTF
CLLCCCKRISGRTCQESVKIKRTNTYATNTTTKESSGSKNGTTVKTSCSSRQRTSGDELAMIDILPV*

Terebratalia transversa orphan neuropeptide GPCR (R3)

LRHLTPLLTLSLVGRAHFFILKSKIIYYWSCRIMSKELLSYLERFNGDKNLTTPMGNADRTYSNIGIIIAYTVIIVISLFGNVLVCQVVYRNKRL
QTVTNIFIVNLAIADILMSSLNIPFTITRLTLDEWVLGSFVCHVFYFIPMVSVYVSTFTLTLIAIDRHQVIVYPLRPKITKRYGVIVLGVIWTLA
ITLAAPFAILAREADIHIVSREVKWCKMDYPHPSVLFHDSITLITISIQYCLPFVIISIMYGRIAKRLWSRGPLGHLTRAQELHHSKTKKKSIRM
LVIVVCIFGLCWLPLNLYHILTDFHTDKLTFRHNSKAFIACHWLAFSSVGYNPFVYCWLNDAFRKEVKHILQCSRKDDSKIHPRGADKKKQQPSL
TTRRTSSIRSTYISSSKVKKDTDIQGKISPDYQGDIDMQDKISPDYQQLTAMLQQDVPMRDINQLTNANQYSERAYPKAMQRDSESISPDERSLI
EALRGAPHASEEDLDDIL*

Terebratalia transversa orphan neuropeptide GPCR, related to P. dumerilii NpY-4 and insect FMRFa receptor (R4)

```
MTCLRTMLENTSPTGKLSMSREITNMTAGLTGVNQTYGNMSVCGHIPDPSTDIIMFQFIAWGIIGSILVLGGCVGNILAIIVLNRHSMGTFTSTY
LSALAIFDTILLLCFLFSFSLPTIWNITWDSTYIDIIYPKMHLVIYPLTLISQQCTIYVTVAFTIQRYCAINWPLKRNKCLLSSRTQALIVITIL
ILGSVIYNSPRMIEFTFYQCYSLQTNQVLQKIVPSEFGSDPTFRKVYHIYLFISVIFMVPFLVLVIFNTLLWLAVRRSKKLQIQKASTVKENNIT
IMLIAIVVVFLICQILPIADNIFMVTLSTATLNNNKYIKFTTISNLMVALNSSINFILYCMFGQRFRQIFLNLFCCKELNINFEGSRSIRWTRLS
SFRSTLRDNKDGGNQPLRDNKDGGNQ
```

Terebratalia GPCR cloning primer:

[Non-gene specific adapters with restriction enzyme sites are given in brackets]

R1:
Forward: [ACAATAGAATTCCGCCACC]ATGGCAGCTGCAAAAGAGTTTC
Reverse: [ACAATAGCGGCCGC]TAATCCCAGACAGAATGCTACCC (includes partial 3′ UTR)

R2:
Forward: [ACAATAGGATCCCGCCA]CCATGTTTTCTGCAATCAC
Reverse: [ACAATAGCGGCCGC]GGCCTTGTTGAATTCTTGT (includes partial 3′ UTR)

R3:
Forward: [ACAATAGAATTCCGCCACC]ATGTCAAAGGAACTACTGAGTTACTT
Reverse: [ACAATAGCGGCCGC]CCTGCACATCAGATAGATGA (includes partial 3′ UTR)

R4:
Forward: [ACAATAGGATCCCGCCACC]ATGATAGAGAATACATCACCTACGG
Reverse: [ACAATAGCGGCCGC]CTACACAAGTAATTTACCCTTGG

Accession numbers of neuropeptide receptor reference sequences

[Xboc.rna.tri.15475.1, *X.bocki*, in transcriptome SRX1343818] [Locus_45236.0_Transcript_1/0, *N.westbladi*, in transcriptome SRX1343819] [Msti.rna.tri.15359.1, *M.stichopi*, in transcriptome SRX1343814] [Msti.rna.tri.31113.1, *M.stichopi*, in transcriptome SRX1343814] [Msti.rna.tri.31092.1, *M.stichopi*, in transcriptome SRX1343814] [Locus_51813.1_Transcript_3/0, *H.spinulosa*, in transcriptome SRX1343820] [AKQ63075.1, *P. dumerilii*, Luqin receptor, deorphanized] [AKQ63063.1, *P.dumerilii*, FMRFamide receptor, deorphanized] [O44426, *L. stagnalis*, Luqin receptor, deorphanized] [P92045, *L.stagnalis*, Lymnokinin receptor, deorphanized] [P49146, *H.sapiens*, Neuropeptide Y receptor type 2, deorphanized] [P97295, *M.musculus*, Neuropeptide Y receptor type 2, deorphanized] [Q9ERC0, *R.norvegicus*, Neuropeptide Y/peptide YY-Y2 receptor, deorphanized] [P29371, *H.sapiens*, Neuromedin-K receptor, deorphanized] [P47937, *M.musculus*, Neuromedin-K receptor, deorphanized] [P16177, *R.norvegicus*, Neuromedin-K receptor, deorphanized] [FBpp0076853, *D.melanogaster*, Leucokinin receptor, deorphanized] [FBpp0084470, *D.melanogaster*, RYamide receptor, deorphanized] [FBpp0081791, *D.melanogaster*, Tachykinin receptor 1, deorphanized] [FBpp0084873, *D.melanogaster*, Tachykinin receptor 2, deorphanized] [ELT88896, *C.teleta*] [XP_009016737, *H.robusta*] [XP_009054576, *L.gigantea*] [XP_009060043, *L.gigantea*] [XP_005090267, *A.californica*] [EKC27293, *C.gigas*] [XP_009027087, *H.robusta*] [XP_007899584, *C.milii*] [XP_009054574, *L.gigantea*] [BAO01094, *N.lugens*] [XP_002730513, *S.kowalevskii*] [XP_002596257, *B.floridae*] [XP_002734699, *S.kowalevskii*] [XP_002738788, *S.kowalevskii*] [XP_002731479, *S.kowalevskii*] [XP_002742045, *S.kowalevskii*] [XP_002596255, *B.floridae*] [NP_001161681, *S.kowalevskii*] [XP_002732003, *S.kowalevskii*] [XP_006812800, *S.kowalevskii*] [XP_009060304, *L.gigantea*] [XP_003700723, *M.rotundata*] [XP_002732001, *S.kowalevskii*] [NP_001161604, *S.kowalevskii*] [NP_001098693.1, *T.rubripes*] [XP_001342488.2, *D.rerio*] [XP_009064591.1, *L.gigantea*] [XP_009067028.1, *L.gigantea*] [XP_009050865.1, *L.gigantea*] [XP_009064514.1, *L.gigantea*] [ELT99672.1, *C.teleta*] [XP_009017792.1, *H.robusta*] [XP_009017796.1, *H.robusta*] [XP_009062052.1, *L.gigantea*] [XP_008498708, *C.anna*] [Q1ACB1, *O.mykiss*] [F1R5V3_DANRE, *D.rerio*] [E9HAW0_DAPPU, *D.pulex*] [G3X054_SARHA, *S.harrisii*] [G1NS97_MELGA, *M.gallopavo*] [J9JKR1_ACYPI, *A.pisum*] [B3XXN5_BOMMO, *B.mori*] [B4MM03_DROWI, *D.willistoni*] [K1PQW2_CRAGI, *C.gigas*] [B3XXN2_BOMMO, *B.mori*] [H3ILX9_STRPU, *S.purpuratus*] [H3ILY0_STRPU, *S.purpuratus*] [H9K8U7_APIME, *A.mellifera*] [Q8VHD7_RAT, *R.norvegicus*] [G1TPU6_RABIT, *O.cuniculus*] [K1Q8V2_CRAGI, *C.gigas*] [D6WD17_TRICA, *T.castaneum*] [Q6AWE5_DROME, *D.melanogaster*] [I4IY86_TAKRU, *T.rubripes*] [F1R3V0_DANRE, *D.rerio*] [F7E6B1_MONDO, *M.domestica*] [B4JUW2_DROGR, *D.grimshawi*] [Q94736_STOCA, *S.calcitrans*] [E9FUQ7_DAPPU, *D.pulex*] [Q8T8D1_UREUN, *U.unicinctus*]
